# Persistent Exposure to Mycoplasma Induces Malignant Transformation of Human Prostate Cells

**DOI:** 10.1371/journal.pone.0006872

**Published:** 2009-09-01

**Authors:** Kazunori Namiki, Steve Goodison, Stacy Porvasnik, Robert W. Allan, Kenneth A. Iczkowski, Cydney Urbanek, Leticia Reyes, Noboru Sakamoto, Charles J. Rosser

**Affiliations:** 1 Department of Urology, The University of Florida, Gainesville, Florida, United States of America; 2 Department of Surgery, The University of Florida, Jacksonville, Florida, United States of America; 3 Department of Pathology, The University of Florida, Gainesville, Florida, United States of America; 4 Department of Pathology, The University of Colorado, Aurora, Colorado, United States of America; 5 Department of Veterinary Pathology, The University of Florida, Gainesville, Florida, United States of America; Max Planck Institute for Infection Biology, Germany

## Abstract

Recent epidemiologic, genetic, and molecular studies suggest infection and inflammation initiate certain cancers, including those of the prostate. The American Cancer Society, estimates that approximately 20% of all worldwide cancers are caused by infection. Mycoplasma, a genus of bacteria that lack a cell wall, are among the few prokaryotes that can grow in close relationship with mammalian cells, often without any apparent pathology, for extended periods of time. In this study, the capacity of *Mycoplasma genitalium*, a prevalent sexually transmitted infection, and *Mycoplasma hyorhinis*, a mycoplasma found at unusually high frequency among patients with AIDS, to induce a malignant phenotype in benign human prostate cells (BPH-1) was evaluated using a series of *in vitro* and *in vivo* assays. After 19 weeks of culture, infected BPH-1 cells achieved anchorage-independent growth and increased migration and invasion. Malignant transformation of infected BPH-1 cells was confirmed by the formation of xenograft tumors in athymic mice. Associated with these changes was an increase in karyotypic entropy, evident by the accumulation of chromosomal aberrations and polysomy. This is the first report describing the capacity of *M. genitalium* or *M. hyorhinis* infection to lead to the malignant transformation of benign human epithelial cells and may serve as a model to further study the relationship between prostatitis and prostatic carcinogenesis.

## Introduction

Mycoplasma, a class of bacteria that lack a cell wall, are among the few prokaryotes that can grow in close relationship with mammalian cells, often without any apparent pathology, for extended periods of time [1]. Due to the fastidious nature of mycoplasma, it is often difficult to isolate the organism from human specimens or the study implements a molecular amplification approach for its detection [2]. As a consequence, many mycoplasmal infections may go unreported. Nevertheless, *M. genitalium* has been identified as the cause of non-gonococcal urethritis in approximately 15–22% of symptomatic men [3]–[5]. In fact, in a sexually transmitted disease clinic in a major US metropolitan area, the presence of *M. genitalium* was more prevalent than *Neisseria gonorrhea* but less prevalent than *Chlamydia trachomatis* [6]. Several studies have also linked *M. genitalium* with chronic persistent prostatitis [7], [8].

Recent epidemiologic, genetic, and molecular studies suggest that infection and inflammation initiate certain cancers, including prostate cancer [9]–[12]. According to the American Cancer Society, approximately 20% of all worldwide cancers are caused by infectious agents [13]. Infectious agents may directly induce tumorigenesis through viral or bacterial protein products that have oncogenic effects or indirectly through a local chronic progressive inflammatory response [14], [15]. Although the mechanisms by which viruses initiate carcinogenesis is being elucidated [12], there is a paucity of information regarding the contributory role of bacterial infection in prostate cancer.

Previously, our laboratory has provided evidence of a correlation between mycoplasma protein activity and malignant potential. Specifically, we demonstrated that recombinant *M. hyorhinis* protein p37 enhanced the invasiveness of two prostate carcinoma and two melanoma cell lines in a dose-dependent manner *in vitro*. These effects could be completely blocked with a neutralizing antibody to p37 [16]. In a separate study, recombinant *M. hyorhinis* p37 induced a more malignant phenotype in prostate cancer cells PC-3 and DU145 as demonstrated by induction of anaplasia and increased migration. Furthermore, these cells showed differential expression of genes involved in cell cycle, signal transduction and metabolism [17]. Taken together, these studies support an association between mycoplasma infection and aggressive cellular phenotype.

In the current study, we investigated the *in vitro* and *in vivo* effects of chronic mycoplasmal infection on a benign human prostate cell line, BPH-1. We demonstrated significant phenotypic and karyotypic changes that ultimately resulted in the malignant transformation of benign BPH-1 cells.

## Results

### Experimental plan

**Figure 1:**
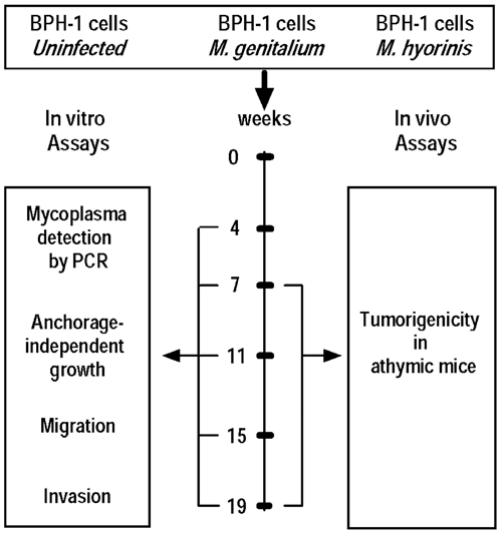
Experimental plan. Benign human prostate BPH-1 cells were infected with the mycoplsmas *M. genitalium* or *M. hyorhinis* and maintained in continuous tissue culture for 20 weeks. Uninfected BPH-1 cells were cultured in parallel for comparison. At weeks 4, 7, 11, 15 and 19, mycoplasmal infection status was confirmed by PCR, and a series of *in vitro* assays were performed. *In vitro* assays included anchorage-independent growth in soft agar, and migration and invasions assays using modified Boyden chambers. At weeks 7 and 19, infected and uninfected BPH-1 cells were inoculated into athymic mice to assess tumorigenicity

BPH-1 cells were infected with *M. genitalium* or *M. hyorhinis* and maintained in continuous culture for 20 weeks. Uninfected BPH-1 cells were cultured in parallel for comparison. At weeks 4, 7, 11, 15 and 19, mycoplasmal infection status was confirmed by PCR, and a series of *in vitro* assays were performed (Figure 1). At weeks 7 and 19, infected and uninfected BPH-1 cells were inoculated into athymic mice to assess tumorigenicity (Figure 1).

### *In vitro* migration and invasion assays

**Figure 2:**
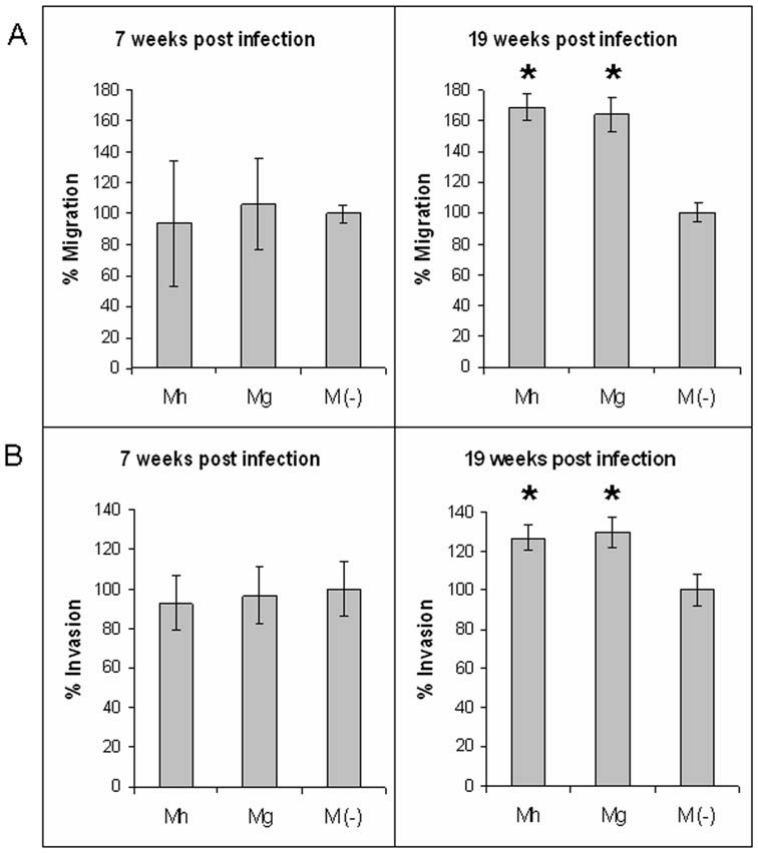
*In vitro* invasion and migration. BPH-1 cells with and without *M. genitalium* (Mg) or *M. hyorhinis* (Mh) infection were tested for migration (A) and invasion (B) potential at weeks 7 and 19 post-infection. Both *M. genitalium* and *M. hyorhinis* infected BPH-1 cells had increased migration and invasion rates at week 19. Data presented are means of triplicates, and the error bars represent the standard error of the mean. *, p<0.05 versus no mycoplasma control (M-).

We have previously published that in a dose-dependent manner, p37 increases tumor cell migration and invasion in both PC-3 and DU145 cancer cells [16], [17]. To investigate whether this phenomenon can be replicated with mycoplasma, we evaluated the migratory and invasive potential of BPH-1, with or without the presence of *M. hyorhinis* or *M. genitalium*, over the course of the 19 weeks of infection. As shown in Figure 2, a statistically significant increase in migration and invasion was evident for BPH-1 cells infected with *M. hyorhinis* and with *M. genitalium* (*p*<0.05) 19 weeks after initial infection. This response was not evident at 7 weeks after inoculation (Figure 2).

### Anchorage Independent Growth

**Figure 3:**
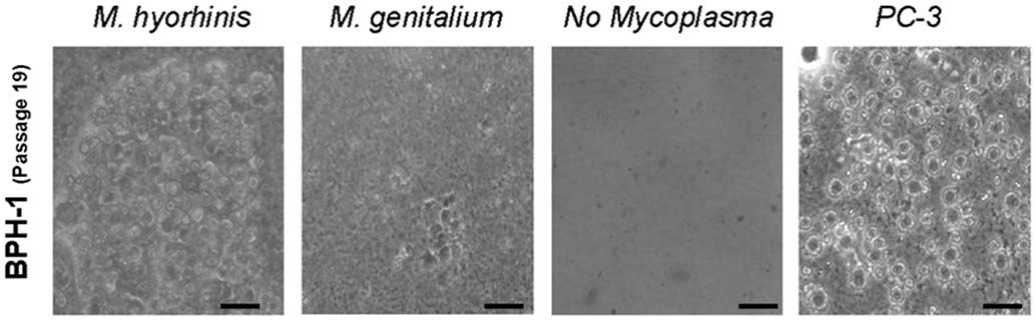
Anchorage-independent growth. BPH-1 cells infected with *M. genitalium* (Mg) or *M. hyorhinis* (Mh) were tested for the ability to grow in soft agar. Persistent infection for 19 weeks with either mycoplasma induced anchorage-independent growth of BPH-1 cells. The PC-3 prostate cancer cell line was used as a positive control. (10 micrometer scale bar  = 2 mm)

Uninfected and mycoplasmal infected BPH-1 cells in culture from passages 7 and 19 were investigated for their capacity to form anchorage-independent colonies in soft agar, a property associated with malignant cell transformation. In parallel with the capacity of cells in culture to migrate and invade, *M. hyorhinis* or *M. genitalium* infected BPH-1 cultures gained the capacity to grow and form colonies in soft agar at 19 weeks after infection (Figure 3). BPH-1 cells infected for only 7 weeks did not exhibit anchorage independent growth. Uninfected BPH-1 cells from week 19 of parallel cutlure were also unable to form colonies in soft agar (Figure 3). The anchorage-independent human prostate cancer cell PC-3, served as a positive control in these experiments (Figure 3).

### Prolonged mycoplasma infection induces karyotypic entropy in BPH-1 cells

**Figure 4:**
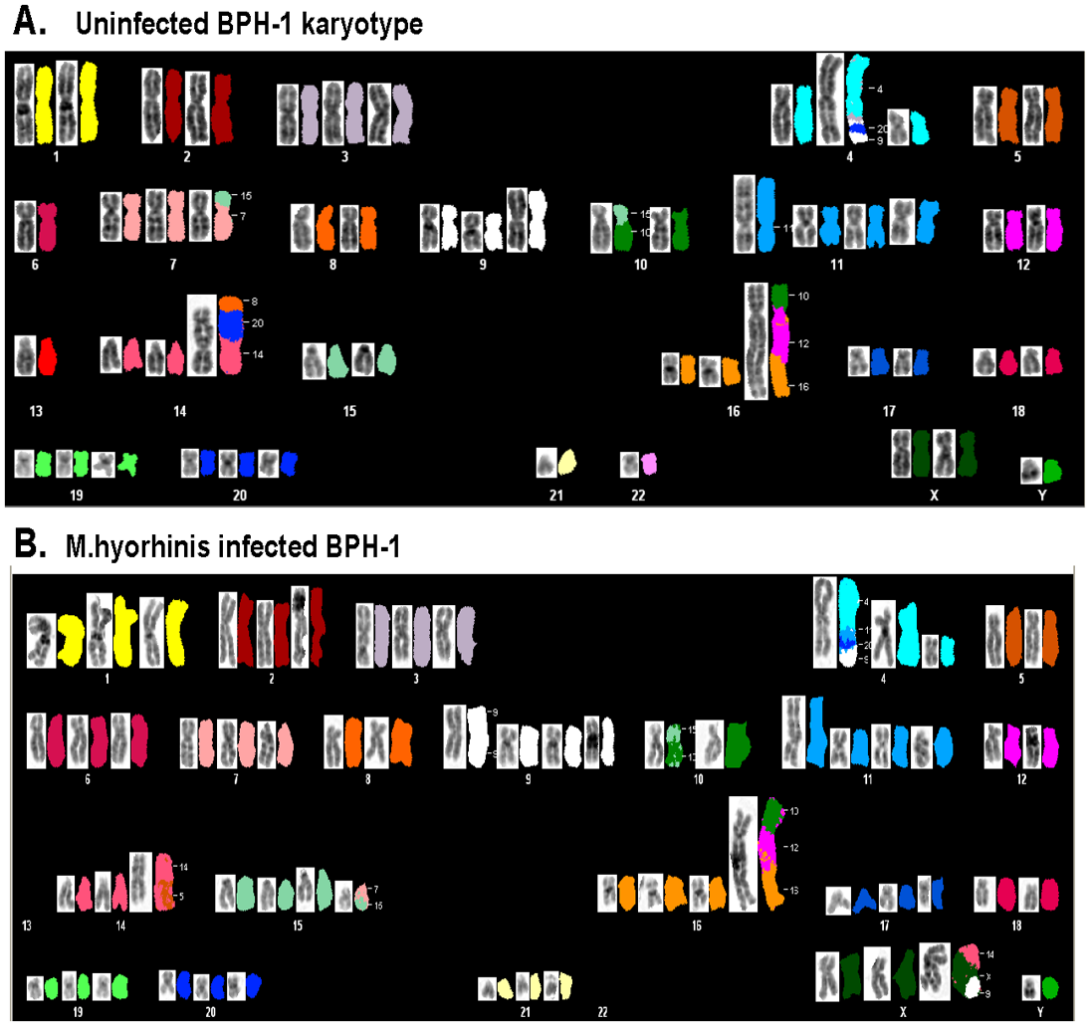
Cytogenetic analysis of mycoplasma infected BPH-1 cells. Metaphase chromosomal complement was revealed by spectral karyotyping (SKY) and counter-staining with DAPI. Representative metaphase cells after SKY classification are shown. A, uninfected BPH-1 cells. B, BPH-1 cells after 19 weeks *M. hyorhinis* infection. Composite for each chromosome shows, left to right, inverted DAPI-stained image, and spectral imaging color representation. Chromosome number is shown below image groupings, numbers alongside the derivative chromosomes indicate the origin of the translocated material. *M. hyorhinis* exposed BPH-1 cells were noted to harbor trisomy of chromosomes 1, 2, 6, 7, 15–18, and 21, in addition to loss of chromosome 13.

The accumulation of chromosomal abnormalities that affect the balance of critical genes involved in the control of cellular growth, differentiation and survival is thought to drive the multistep process of cancer development and progression. To investigate whether gross structural chromosomal changes were associated with the phenotypic changes observed in the BPH-1 prostate cell line model, we performed a detailed molecular cytogenetic evaluation of BPH-1 cells subjected to 19-week mycoplasma infection and compared the karyotype with uninfected cells. Using spectral karyotyping (SKY), a multicolor technique that enables the visualization of all 48 human chromosomes through fluorescent image analysis [18], [19], we were able to define the karyotype of BPH-1 cells and resolve the identity of marker chromosomes. Metaphase chromosome preparations were made from parallel, exponentially growing cultures (untreated and mycoplasma-infected). Parental BPH-1 cells (Figure 4A) had a modal number of 53 chromosomes (average number of total chromosomes) with relatively few aberrations, including 2 marker chromosomes t(4;11;20;9) and t(10;12;16). The parental BPH-1 karyotype was: *53*, *XXY,+3,del(4q),+t(4;11;20;9),6,+t(7;15),+i(9or18),t(10;15),+11,+add(11p)*,*13, +t(8;20;14),+t(10;12;16),+19,+20,-21,-22*. Conversely, markedly altered karyotypes were observed after prolnged infection with mycoplasma. The representative metaphase spread shown in Figure 4B depicts the variety of rearrangements observed in the *M. hyorhinis* infected BPH-1 cells. These cells retained the 2 major marker chromosomes, t(4;11;20;9) and t(10;12;16), but also gained random marker chromosomes (for example, t(9;X;14). However, the major difference observed in the infected cells was the accumulation of multiple copies of apparently normal chromosomes, i.e., increased copy number of chromosomes. The modal number was 63 (over 53 for uninfected cells described above). Trisomy of chromosomes 1, 2, 6, 7, 15–18, and 21 was typical. Loss of chromosome 13 was also observed in multiple metaphases (Figure 4B). The full karyotype for *M. hyorhinis* infected BPH-1 cells was as follows (most consistent changes in bold): *53-65,XXY, +* ***1,+2*** *,+3,+t(4;20;9),del(4),* ***+6,+7*** *,+t(7;15),+i(9),+9,+t(10;15),+11,add(11),* ***+t(5;11),13x2,+t(14;5),+5,+16*** *,+t(10;12;16),+* ***17,+18*** *,+19,+20,* ***+21*** *,-22x2,+* ***t(9;X;14)*** *.* In similar fashion, BPH-1 cells infected with *M. genitalium* showed multiple chromosomal aberrations. The karyotype for these cells was (most consistent changes in bold): *61-65,XY,+X,* ***+1,+2*** *,+3,+der(4)t(4;11;20* ***;18),+8*** *,+9,* ***der(10;22)t(10;22),*** *+11* ***,+11withins,*** *+der(12)t(10;12;16),+* ***der(14)t(14;*** *),+der(14)t(14;20;8* ***),+15,+16,+17,+*** ** ***i(18),t(16;18)*** *,+19,+20* ***,+21,-*** *22 (data not shown).* Although there were many differences in detail, the overall accumulation of aberrations caused by infection by the two mycoplasma species was similar, and the major change observed was increased polysomy, i.e. the addition of extra, complete chromosomes.

### Tumorigenicity of BPH-1 cells in nude mice

We determined whether mycoplasmal infection could induce the gold standard of malignant transformation, tumorigenicity. Uninfected BPH-1 cells, or BPH-1 infected with *M. hyorhinis* or *M. genitalium* from passage 7 and passage 19 were inoculated subcutaneously in male nude mice. Tumors were monitored twice weekly for 15 weeks and recorded. Only subcutaneous growths >5 mm in diameter were considered to be a tumor. No tumors were evident in any animals inoculated with BPH-1 cells isolated from passage 7. However, infected cells from passage 19 did initiate tumors. Three tumors (43%) developed after *M. genitalium* infected cell inoculation (median tumor size 190±20 mm^3^), and five tumors (63%) developed from *M. hyorhinis* infected cell inoculations (median tumor size 87±10 mm^3^). No tumors developed at sites inoculated with uninfected BPH-1 cells, only small nodules (1–3 mm diameter) were evident in some animals (Table 1).

**Table 1:** Growth of BPH-1 xenograft tumors.

Culture	7 week	19 week
	Tumorigenicity in mice (%)	Tumorigenicity in mice (%)
Uninfected	0/7 (0)	0/7 (0)
*M. genitalium*	0/7 (0)	3/7 (43)
*M. hyorhinis*	0/7 (0)	5/8 (63)

**Figure 5:**
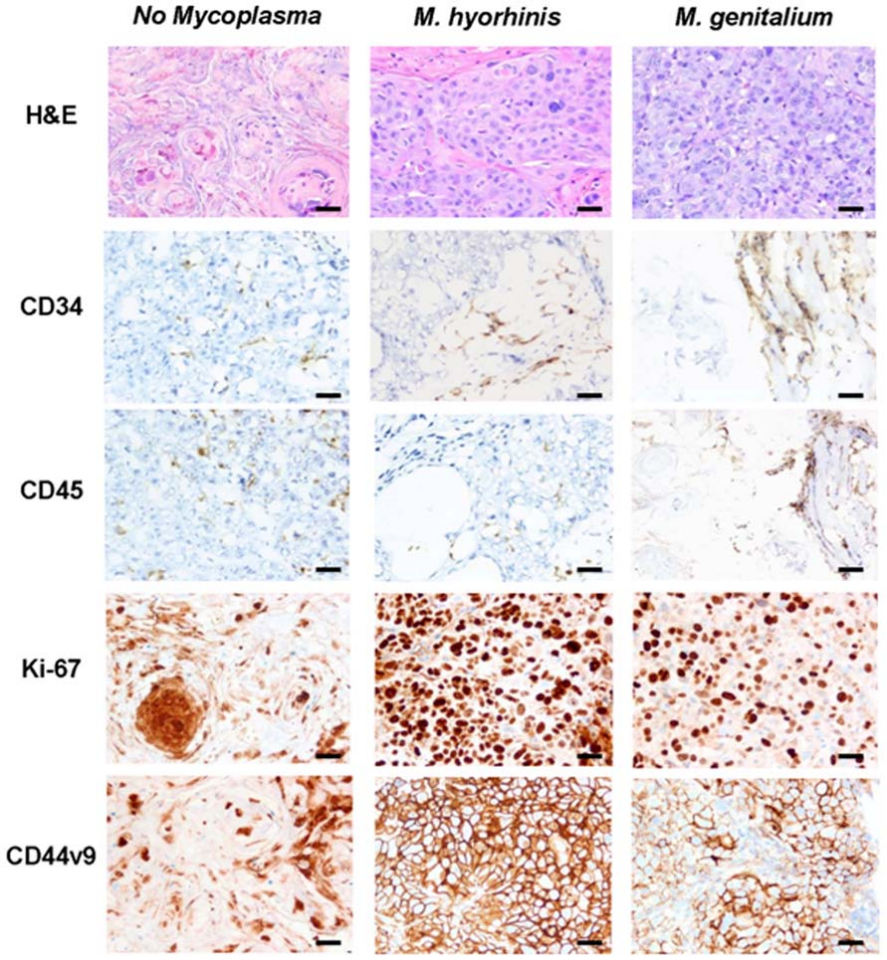
Molecular analysis of human BPH-1 xenograft tumors grown in nude male mice. Tumors generated by mycoplasma infection in BPH-1 cells did not have a typical adenocarcinoma appearance, but a more squamoid appearance (H&E). In addition, uninfected BPH-1 cells from passage 19 grown subcutaneously in nude mice were less angiogenic (CD34) and harbored more leukocytes (CD45) than did xenograft tumors from BPH-1 cells infected with *M. hyorhinis* or *M. genitalium*. Only mycoplasma infected BPH-1 cells formed xenograft tumors. Expression of markers of malignancy (CD44v9 and Ki-67) were revealed using immunohistochemistry. (10 micrometer scale bar  = 2 mm)

To confirm the composition of tumors/nodules, xenograft tissues were resected, stained with hematoxylin and eosin, and histologically examined. The uninfected BPH-1 cells present in small nodules had uniform round nuclei and a mosaic pattern of growth without atypia or infiltration. BPH-1 cells infected with *M. genitalium* grew in solid nests with indistinct cell borders and had nuclear atypia and active mitosis. Furthermore, invasion of these cells into skeletal muscle was observed. BPH-1 cells infected with *M. hyrohinis* grew in solid nests with minimal intervening stroma and had nuclear atypia and active mitosis. Tumors generated by mycoplasma infection in BPH-1 cells did not have the typical glandular appearance of adenocarcinoma but instead had more features of squamous differentiation (Figure 5). Previous studies have documented the inability of unmanipulated BPH-1 cells to grow as xenograft tumors in athymic mice [20].

To further investigate the composition of BPH-1 tumors, immunohistochemical analyses were used to monitor the expression of CD34, CD45, CD44 and Ki-67. CD34 immunostaining was utilized to reveal intratumoral vasculature. BPH-1 tumors exposed to mycoplasma organisms demonstrated increased vascularity compared to the small nodules produced by BPH-1 cells not exposed to mycoplasma (Figure 5). CD45 immunostaining was employed to determine whether uninfected BPH-1 nodules were influenced by an accumulation of lymphoid cells. Figure 5 clearly demonstrates that the bulk of the mycoplasma infected tumors were devoid of lymphocytes. Conversely, subcutaneous masses generated from uninfected BPH-1 cells contained more lymphocytes (Figure 5). In addition, the weak, focal CD44 variant 9 expression in the tissue of the uninfected BPH-1 nodules confirms the lack of BPH-1 outgrowth in these nodules. In tumors derived from BPH-1 infected with *M. genitalium* or *M. hyorhinis*, there was strong membranous expression of this cell-surface tumor cell marker (Figure 5). Ki-67, a marker of proliferation, was negative in uninfected BPH-1 cell nuclei, but was positive in over 50% of the nuclei of cells infected with *M. genitalium* or *M. hyorhinis* (Figure 5). Previously reported malignant transformation of BPH-1 cells by CAF or hormone treatments also resulted in a significant increase in the proliferative index as revealed by an increase in Ki-67 immunoreactivity [21].

## Discussion

This is the first report describing the potential of mycoplasma infection to lead to malignant transformation of benign human prostate cells in cell culture and xenografts. Persistent infection of BPH-1 for 19 weeks induced increased migration/invasion, the acquisition of anchorage-independent growth, karyotypic entropy, and finally, malignant transformation proven by the formation of xenograft tumors in immune-compromised mice. BPH-1 cell growth is normally anchorage-dependent and does not form tumors in immunocompromised mice. Thus, we have developed a model that may mimic aspects of the clinical progression of human prostate cancer, and which can be applicable to the further study of the link between infection and malignancy. While immortalization or transformation of mammalian cells has been shown with some other mycoplasma [22], [23], to our knowledge, this is the first time mammalian cell transformation has been achieved by infection with either *M. genitalium* or *M. hyorhinis*.

Mycoplasma can cause a wide variety of diseases in mammals, but most often they colonize respiratory and urogenital tracts without immediate clinical significance [1], [6]. Owing to their limited biosynthetic capabilities, most mycoplasma are obligate parasites, but by associating with mammalian cell membranes they may evade immune surveillance, and, therefore, they can persist chronically. Prolonged infections with mycoplasma of even seemingly low virulence could affect the phenotype of mammalian host cells [23]–[25]. It is conceivable that chronic infection by certain mycoplasma may increase host cell vulnerability to transformation events and promote tumor growth of mammalian cells.

Of the two mycoplasma employed in this study, *M. genitalium* is an established human pathogen already linked to urethritis [3]–[5], [26]–[28] and prostatitis [7], [8], [29]. *M. hyorhinis* is better known as a porcine pathogen, but its status as the mycoplasma strain most commonly infecting laboratory cell lines [1] implies that it can thrive among human cells. The strongest link between *M. hyorhinis* and human cancer was reported recently by Huang et al., who used a monoclonal antibody against the unique *M. hyorhinis*–specific protein p37 to detect mycoplasma infection in over 600 carcinoma tissues from a variety of organs. The study indicated that up to 56% of gastric carcinoma and 55% of colon carcinoma biopsies were positive for *M. hyorhinis* [30].

In our own work, we have previously demonstrated that treating PC-3 and DU145 human prostate with recombinant *M. hyorhini*s protein p37 results in increased proliferation, migration and morphological changes, and significant perturbation in gene expression profile [16], [17]. We have recently elucidated the crystal structure of the *M. hyorhinis* p37 protein and refined it to 1.6 Å resolution. *Mh*-p37 has high homology to bacterial ABC transporters, but we unexpectedly found that the protein binds thiamine pyrophosphate, or vitamin B1 [31], [32]. Investigations into the role of *Mh*-p37 are ongoing, but understanding the function of this protein may lead to the development of anti-mycoplasmal agents, which given the findings of this study, may have clinical utility in the field of prostate health.

The capacity of mycoplasma to induce malignancy in murine cells has been previously demonstrated, however, murine cells are known to be much more vulnerable than human cells to transformation by a variety of stress-inducing events. Chronic infection with *M. fermentans* or *M. penetrans* transformed C3H mouse embryo cells. This mycoplasma-mediated oncogenic process had a long latency and showed multistage progression. After 18 weeks of infection, C3H cells revealed prominent chromosomal changes, and were able to form tumors in animals [23]. A study using 32D cells, a murine myeloid cell line, showed that infection by *M. fermentans* or *M. penetrans* for only 4 to 5 weeks induced malignant transformation of these cells. Transformed 32D cells grew autonomously and quickly and ultimately formed tumors when injected into nude mice [22].

Studies of prolonged mycoplasmal infection of human cells have been rare. Zhang et al showed that *Mycoplasma fermentans* infection of at least 6 weeks duration prolonged the survival of human peripheral blood mononuclear cells (PBMCs) from healthy blood donors in culture, and markedly enhanced the rate of EBV-positive B lymphocytes to undergo immortalization, but not transformation [33]. Without specific supplemental growth factors, human PBMCs normally die rapidly in culture. To our knowledge, the only other transformation of human cells by mycoplasma infection used BEAS-2B, an immortalized human bronchial epithelial cell line. These cells were immortalized by infection with an adenovirus 12-SV40 virus hybrid (Ad12SV40) and express the SV40 T antigen. BEAS-2B cells differentiate and die when they become confluent in culture, and transform spontaneously after 32 passages in subconfluent culture [34]. After prolonged infection (44 days) with *Mycoplasma arginini,* BEAS-2B cells were observed to overcome contact inhibition and to gain the capacity to grow when suspended in soft agar. After 12 weeks of exposure to *M. arginini*, BEAS-2B cells were able to form tumors in athymic nude mice.

The immortalized BPH-1 cell line was derived from primary cultures of prostatic epithelial cells by introducing SV40T antigen [20]. SV40T inactivates both p53 and Rb, thus negating two important tumor suppressor pathways [34], however, as is the case with BPH-1, many SV40T-expressing cells are not tumorigenic [35], [36]. The BPH-1 cell line is known to be susceptible to malignant transformation when exposed to appropriate stimulation. Wang et al. were able to induce formation of invasive BPH-1 tumors in the renal capsule of athymic mice through recombination with rat urogenital sinus mesenchyme (UGM) [37]. The same research group has also shown that BPH-1 can be induced to form xenograft tumors when combined with carcinoma associated fibroblasts (CAFs) [21]. We show in this study that untreated BPH-1 cells can survive in a murine host. It has been previously shown that viable BPH-1 cells can be recovered from the graft site up to 1 year later [21], hence, in sum they meet the criteria of being ‘nontumorigenic’. BPH-1 cells can however be considered to be genetically initiated, in that they have lost their aneuploidy checkpoint mechanism, presumably attributable to the expression of SV40T, and the accumulated chromosomal material increases the probability of DNA damage accumulation. Further insult or stress by an unknown mechanism may render them susceptible to further genetic damage and to progression along a pathway to malignancy. The mechanism by which such common genetic changes could be induced by mycoplasmal infection environment is at present unclear, but aneuploidy is one of the most obvious differences between normal and cancer cells, and accordingly, has been proposed to be a pivotal mechanism in cancer progression [38]. Given the genomic instability associated with aneuploidy, it is logical that cells have evolved mechanisms to prevent either the proliferation or survival of aneuploid cells. Indeed, it has long been observed that aneuploid cells often undergo a p53-dependent cell cycle arrest in the G1 phase following cytokinesis failure [39]. As described above, BPH1 cells have a compromised p53 function.

Our study revealed that while infection of BPH-1 cells was transformative, the same mycoplasma could not influence the phenotype of human CCL-123, a human fibroblast cell line that has not been genetically manipulated in any way (data not shown). CCL-123 cells seemed oblivious to the presence of mycoplasma infection with either species; the cells exhibited no change in culture, and entered senescence at passage 11–13 regardless of mycoplasma infection status. This supports the notion that mycoplasma are more likely to exacerbate host cell progression to a malignant phenotype, rather than initiate cellular transformation. It is possible that mycoplasma can initiate the process over extended periods of infection, but given the limited lifespan in culture of ‘normal’ human cell lines such as CCL-123, this hypothesis is difficult to test.

Prostate cancer, the most common visceral malignancy in American men, will be diagnosed in approximately 186,320 men and result in 28,660 deaths in 2006 [40]. Accordingly, epidemiologic studies show that chronic inflammation is associated with an increased risk of malignancy. The strongest associations revolve around Epstein-Barr virus with lymphoma, hepatitis B with liver cancer and Schistosomal infection with bladder cancer [41], [42]. Recently, research has linked prostatitis, i.e., infection of the prostate, to the development of prostate cancer. While the prevalence of asymptomatic prostatitis is not known, symptomatic prostatitis occurs in 9–10% of men between 40 and 79 years of age. In most cases, no causative infectious organism can be identified, so it is difficult to link symptomatic or asymptomatic prostatitis with prostate cancer [7]. Mycoplasma organisms are notoriously difficult to grow in standard culture condition and thus are not easily detected by routine microbiologic techniques. The fact that mycoplasma organisms have the capacity to be internalized into human host cells creates another obstacle to detecting mycoplasma [43]. To further investigate a linkage between mycoplasma infection and prostate cancer, we are currently performing quantitative screening of a large cohort of benign and cancerous human prostate tissue samples for the presence of *M. genitalium* and *M. hyorhinis*.

Based on three studies to date, viral involvement seems to be associated with mycoplasma-induced human cell perturbation. Mycoplasma have now been shown to immortalize or transform EBV-positive PBMCs, SV40T expressing BEAS-2B cells, and now SV40T-expressing BPH-1 cells. This implies that concomitant infections with low-virulence viruses and mycoplasma may synergistically induce accelerated transformation to malignancy. Given the prevalence of nonpathogenic viral infections in humans, it is entirely feasible that this double-infection could occur at frequencies similar to those of cancer prevalence. It is interesting to note that many Americans who took the Salk polio vaccine were exposed to SV40. It has been estimated that 10–30 million of the 98 million people who received a polio shot during the period 1955–1963 actually received a vaccine that was contaminated with SV40. In addition, about 10,000 volunteers who received an experimental oral polio vaccine (OPV) between 1959–1961 may have been exposed to SV40. Evidence to date indicates that after 1963, all vaccines on the U.S. market were free of SV40, but an epidemiological study to investigate potential association of individuals receiving SV40-contaminated vaccines with disease in organs susceptible to chronic bacterial infection could be revealing.

We have demonstrated the potential of *M. genitalium* and *M. hyorhinis* to transform benign human prostate cells via a multi-stage process over an extended period of exposure. Ultimately, persistent mycoplasma infection over 19 weeks resulted in BPH-1 cells gaining anchorage-independent growth in culture and the capacity to form tumors in immunocompromised mice. Associated with the acquired tumorigenicity was an accumulation of chromosomal changes, typified by an increase in aneuploidy. These observations suggest that *M. genitalium* and *M. hyorhinis* infection can recapitulate genomic and phenotypic changes most often associated with progression to malignancy. Further studies into the mechanisms involved in this phenomenon are required, but the idea that mycoplasma infection can exacerbate, or perhaps even initiate human prostate malignancy may stimulate new thinking on how we prevent, diagnose and treat prostate cancer.

## Methods

### Cell Lines and Culture

Human benign prostate cell line, BPH-1, and human benign fibroblast cell line, CCL-123 were obtained from the American Type Cell Culture (Manassas, VA). Cells were maintained in RPMI media supplemented with 5% fetal bovine serum, 4.5 g/l glucose, 4 mM L-glutamine, 100 units/ml penicillin and 100 µg/ml streptomycin. All cells were incubated at 37°C in a humidified atmosphere of 5% CO_2_ in air. All culture media were purchased from Invitrogen (Carlsbad, CA).

### Mycoplasma preparation and culture

*Mycoplasma genitalium* was a gift from Dr. Joel Baseman (University of Texas Health Science Center, San Antonio, TX). A stock solution of *M. genitalium* was prepared by growing the microorganism to early log phase in SP4 broth, and stored frozen at −80°C. *Mycoplasma hyorhinis* SK76, was a gift from Dr. Kim Wise (University of Missouri, Columbia, MO). A stock solution of *M. hyorhinis* was grown to early log phase in Frey's broth, and stored frozen at −80°C. Cultures for CFU determination of inoculum were serially diluted 10-fold to 10^−8^ in appropriate broth. Then 20 µl from each sample and its corresponding dilutions (10^−8^) were plated on SP4 or Frey's agar. Agar plates were incubated at 37°C in 5% CO_2_; broth cultures were incubated at 37°C in ambient air. Broth tubes were checked daily for a color change, and the reciprocal of the last dilution to show growth was deemed the color-changing unit (CCU). In order to confirm growth of *M. genitalium* and *M. hyorhinis*, color-changing units (CCU), which provide a relative indication of the amount of microbes present, were determined. Samples were serially diluted tenfold in SP4 or Frey's broth in duplicate. Broth tubes were checked daily for a color change, and the CCU was recorded as the reciprocal of the last dilution to show growth at 21 days. Agar cultures were incubated for at least 5 days before colonies were counted. Human benign prostate BPH-1, and human benign fibroblast CCL-123 cells, previously tested negative by PCR for mycoplasma contamination (PCR Mycoplasma Detction Ste from Takara, Japan), were grown in complete RPMI with 10% fetal bovine serum (FBS), without antibiotics. Cell densities in each flask were reduced to approximately 0.05×10^5^ cells per flask by passaging without trypsin (Versene) on a weekly basis, when cell density approached confluence. Cell cultures typically grew from a density of 10^4^ cells per ml at day 1 to 10^6^ cells per ml at day 7.

Freshly seeded monolayer cultures of BPH-1 and CCL-123 cells were prepared in quadruplicate, with cell density of 0.5×10^5^ per 25 cm^2^ flask. One flask of each cell line was inoculated with 0.01 ml sterile SP4, 4×10^3^ CFU of *M. genitalium* (0.01 ml), 0.01 ml of sterile Frey's or 4×10^3^ CFU of *M. hyorhinis* (0.01 ml). After 24 hours, cell culture medium from each flask was exchanged with fresh sterile complete RPMI/FBS. Subsequent medium changes were performed every 72 or 96 hours until cell harvest. *M. genitalium*, and *M. hyorhinis* infection of inoculated cells was confirmed by PCR. Briefly, the following primers were used for PCR: Mh sense primer 5′- ACACCATGGGAGCTGGTAAT-3′ and antisense primer 5′- CTTCTCGACTTTCAGA-3′ and Mg sense primer 5′- AGTTGATGAAACCTTAACCCCTTGG-3′ and antisense primer 5′- CCGTTGAGGGGTTTTCCATTTTTGC-3′. The amplification conditions of Mg PCR consisted of initial denaturation at 95'C for 10 min followed by 32 cycles of denaturation at 95'C for 30 sec, annealing at 60'C for 1 min and elongation at 72'C for 1 min. The amplification conditions of Mh PCR consisted of initial denaturation at 94'C for 1 min followed by 35 cycles of denaturation at 94'C for 30 sec, annealing at 55'C for 2 min and elongation at 72'C for 1 min. BPH-1 and CCL-123 cultures at 7 and 19 weeks post infection were assessed for changes associated with malignant transformation. Unfortunately by week 11, CCL-123 cell viability was markedly reduced and by week 13 the reduction in cell number precluded any further assessment of these cultures *in vitro* or *in vivo*. The growth rate of immortalized BPH-1 cells in culture was unchanged over the 19 weeks of the study. All *in vitro* and *in vivo* analyses were performed on parallel cultures (untreated or infected with *M. hyorhinis, M. genitalium*) originating from the same stock, and were analyzed at the same passage.

#### In vitro cell migration and invasion assays

Migration assays were performed in six-well two-tier invasion chambers (Collaborative Biomedical Products, Bedford, MA, USA), using a protocol similar to that used successfully by others [44]. Polycarbonate membranes were coated with 4 mg/ml growth factor reduced Matrigel (BD Biosciences, San Jose, CA) as described for invasion assays, control inserts (migration only) contained no coating. Uninfected and mycoplasmal infected BPH-1 and CCL-123 cells were added to each insert at a density of 100,000 cells/ml/well in RPMI media. The lower chamber contained RPMI media with 10% FBS as chemoattractant. After incubation in a humidified incubator with 5% CO_2_ at 37°C for 24 hours, the cells on the top of the polycarbonate membrane were removed. The cells attached to the bottom of the membrane were fixed in 100% methanol, stained with LeukoStat Staining kit (Fisher), and counted microscopically. Each assay was performed in triplicate. Comparisons between group means were assessed with the paired Student t-test.

### Anchorage-Independent Cell Growth

Anchorage-independent cell growth was assayed by the capacity of cells to form colonies in soft agar. The technique of examining cloning efficiency of various transformed cells in soft agar was previously described in detail [45]. To assess the cloning efficiency, 1×10^4^ uninfected or mycoplasma infected BPH-1 or CCL-123 cells were plated on Petri dishes in 0.3% agar/media. The presence or absence of colonies was documented.

### Spectral Karyotyping (SKY)

At week 19, only BPH-1 cells were analyzed cytogenetically by Spectral Karyotyping (SKY). Karyotypic data were obtained from 5–10 fully analyzed metaphase cells per cell line. Metaphase spreads were prepared using standard cytogenetic methods, and slides were aged overnight at room temperature. SKY hybridization and image capture/analysis were performed according to the manufacturer's instructions (Applied Spectral Imaging, Carlsbad, CA) as previously described [19,49]. Probes were created by polymerase chain reaction (PCR) amplification using degenerate primers and incorporation of three fluorochromes and two haptens (biotin and digoxigenin) using purified single-chromosome templates. Haptens were detected indirectly using Cy5-conjugated avidin and Cy5.5-conjugated antimouse IgG (Rockland, Gilbertsville, PA). Slides were counterstained with DAPI and mounted with Prolong Antifade solution (Molecular Probes, Eugene, OR). Spectral imaging was achieved using a SpectraCube system (Applied Spectral Imaging) viewed through a 60x objective illuminated by a xenon lamp. Chromosomes were classified with SkyView software (Applied Spectral Imaging). DAPI banding was captured separately and inverted for alignment with spectral representations using SkyView software.

### Tumorigenicity in nude mice

BPH-1 cells were further studied in an *in vivo* setting. Uninfected and mycoplasma infected BPH-1 cells (1×10^6^ in 1 mg/ml Matrigel, Becton Dickinson Labware, Bedford, MA) from passages 7 and 19 were injected subcutaneously into the right flanks of 5- to 6-week-old nude male mice (Harlan-Sprague-Dawley, Indianapolis, IN). Seven to eight mice were in each group. The mice were carefully followed for 15 weeks for tumor formation. At 15 weeks, mice were sacrificed. Full necropsies were performed and histopathology of tumors was documented.

### Immunohistochemical Analysis

Subcutaneous tumors or nodules at the site previously injected with BPH-1 cells were assessed by two independent pathologists (R.W.A., K.A.I.) using histopathologic examination. In addition, a panel of immunohistochemical stains was performed to a) identify areas of angiogenesis in tumors, b) identify lymphoid infiltrate possibly mimicking a tumor, c) identify tumor associated antigens, and d) estimate the cellular proliferative rate in tumors. Angiogenesis and lymphoid infiltrate were assessed by immunostaining utilizing antibodies to CD 34 and CD 45, respectively. CD44v9 served as a marker for prostate malignancy [47], [48].

Frozen tumor sections (8–10 µm thick) were mounted on Superfrost Plus slides (Fisher Scientific, Hampton, NH), fixed in cold acetone for 5 min, and then incubated overnight at 4°C with monoclonal rat anti-mouse CD34 antibody (RAM 34 clone) (1∶100 dilution; BD PharMingen, San Diego, CA), monoclonal rat anti-mouse CD45 antibody (Ly 5 clone) (1∶25 dilution; BD PharMingen, San Diego, CA), or monoclonal anti-CD44v9 antibody (HB-258 hybridoma supernatant, ATCC). In addition, 5 µm thick paraffin sections of xenograft tissues were placed on poly-L-lysine-coated slides. The slides were deparaffinized and treated with 0.3% hydrogen peroxide and deionized water to block endogenous peroxidase activity. The sections were incubated overnight with Ki-67 antibody (dilution 1∶100; Dako, Carpinteria, CA), a marker for cellular proliferation. Specimens were washed 4 times with PBS after antibody incubation, treated with the appropriate dilution of a biotinylated anti-rabbit IgG, anti-mouse IgG, or anti-rat IgG, and then incubated at room temperature for 1 hour. Finally, the slides were counterstained with hematoxylin, dehydrated, and mounted on slides with Permount.

### Statistical Analysis

Migration and invasion assays were analyzed by student t test (paired) and tumor incidence was analyzed by the χ^2^ test. A value of *P<*0.05 was considered significant.
